# Notes by a Family Physician Shootling

**DOI:** 10.51866/mol.246

**Published:** 2022-11-15

**Authors:** Suzane Shiyun Chin

**Affiliations:** 1 MD (UI), FRACGP (Australia), Klinik Kesihatan Bakri, Muar, Johor, Malaysia. Email: suzanecs88@gmail.com

## Preface

This article was written at leisure for the World Family Doctor Day, which fell on 19 May 2022, but the author fell victim to busy weeks and finished the article late.

The author is a young family physician currently approaching the end of her gazettement and has a love for writing cultivated from her long-gone schooldays.

A (very belated) Happy World Family Doctor Day to all comrades fighting the good fight for the betterment of our patients every day.

Quotes courtesy of Sir William Osler and Robert Greene.

**‘A doctor is a student till he dies, once he considers himself not a student anymore, the doctor inside him dies’.**

Over a table of catch-up tea, my non-medical friends would ask me what I specialise in as a doctor. I would say ‘family medicine’, and 90% of the time, they would draw a blank. The common conclusion my friends would incorrectly jump to, much to my amusement, would be the reproductive and infertility subspecialty owing to the word ‘family’.

I would laugh and begin the long story.

Family medicine is currently not as well known a specialty to the public in Malaysia as surgery and general medicine. In countries like Australia and the United Kingdom, family medicine is arguably the most important specialty, given their universal healthcare access and the requirement of an accreditation in the form of a specialist or fellowship training to be able to practice as a general practitioner.

I was not particularly inspired by any figure or incident to undertake the pathway — but the need for constant learning pushed me to pursue a fellowship, and a meddlesome attitude of wanting to know enough across multiple fields motivated me to choose family medicine.

Medicine is a forever-changing landscape, and clinical guidelines are constantly evolving as new therapies are being discovered. As a doctor, one has to learn to keep up — or else, stagnate and compromise patient care.

And in my humble opinion, family medicine, which spans care across various disciplines, is the most enriching knowledge vault of all.

**‘A jack of all trades is a master of none, but oftentimes better than a master of one’.**

Family medicine specialists are whom I consider specialists of integration. It is essential to learn enough knowledge from various fields and bring these disciplines together to manage patients in a holistic manner.

I once had a memorable pregnant patient whom I saw safely all the way to delivery. She had bronchial asthma, gestational diabetes and anaemia brought about by poor spacing. Following her delivery, we had to work together around the financial implications, family planning and her worries. I continued to see her youngest child on follow-ups, with my care extending from when he was in the womb all the way to being an active 3-year-old.

The care of patients in primary care does not end upon discharge unlike that in hospital inpatient care. The management continues for a long time, and in a majority of that time, it extends to other family members as well.

This comprehensive management is what encompasses family medicine and where it derives its name from.

**‘It is much more important to know what sort of a patient has a disease than what sort of a disease a patient has’.**

Family medicine is likely the only unique discipline that equally targets patients’ psychosocial aspects alongside disease pathophysiology. Other than the duties of a treating physician, the family physician becomes a form of confidante and advisor to his or her patient.

Tackling this aspect has been one of the most challenging yet fulfilling aspects in my trade. I learned that there is never a one-size-fits-all method to approach and communicate with patients. The perception of an 80-year-old retiree and that of a 30-year-old lecturer are rarely similar.

There will be patients’ fears that will need addressing, misconceptions that will require careful information-sharing and awareness of diseases that will need to be instilled.

There will be patients whose personal outlooks may impede and worsen their clinical profile, and there may be ones we will fail to empower; we do not stop trying, regardless.

We are privileged to be a part of our patients’ journeys.

**‘One of the first duties of the physician is to educate the masses not to take medicine’.**

There is no practice in the medical field where the slogan ‘Prevention is better than cure’ applies more heavily than in family medicine.

Diseases and complications are already present in most patients presenting to the hospital. In primary care, both the sick and healthy are seen. Health education and screening are an integral part of family medicine, and the activities are wonderfully diverse - from vaccination and cancer screening to preconception care.

I used to joke to my hospital colleagues that in place of 24-hour on-calls, we do door calls. Another distinguishing feature of this specialty is community work, whereby doctors go out into the field and actively involve the public in health education and screening.

**‘Proud to be a family physician’**

Not one patient who walks through my door at any time of the day is the same. Each case is constantly evolving much like a puzzle that changes with each consultation. That is what I love about practicing family medicine.

Whether a patient with diabetes ends up to be admitted for a target-organ complication years down the road or ends up aging well with great metabolic control largely lies on what we strive to do for him or her today.

Family medicine is a beautiful and wholesome branch of specialty — one that I am proud to be a shootling of, amongst many.

